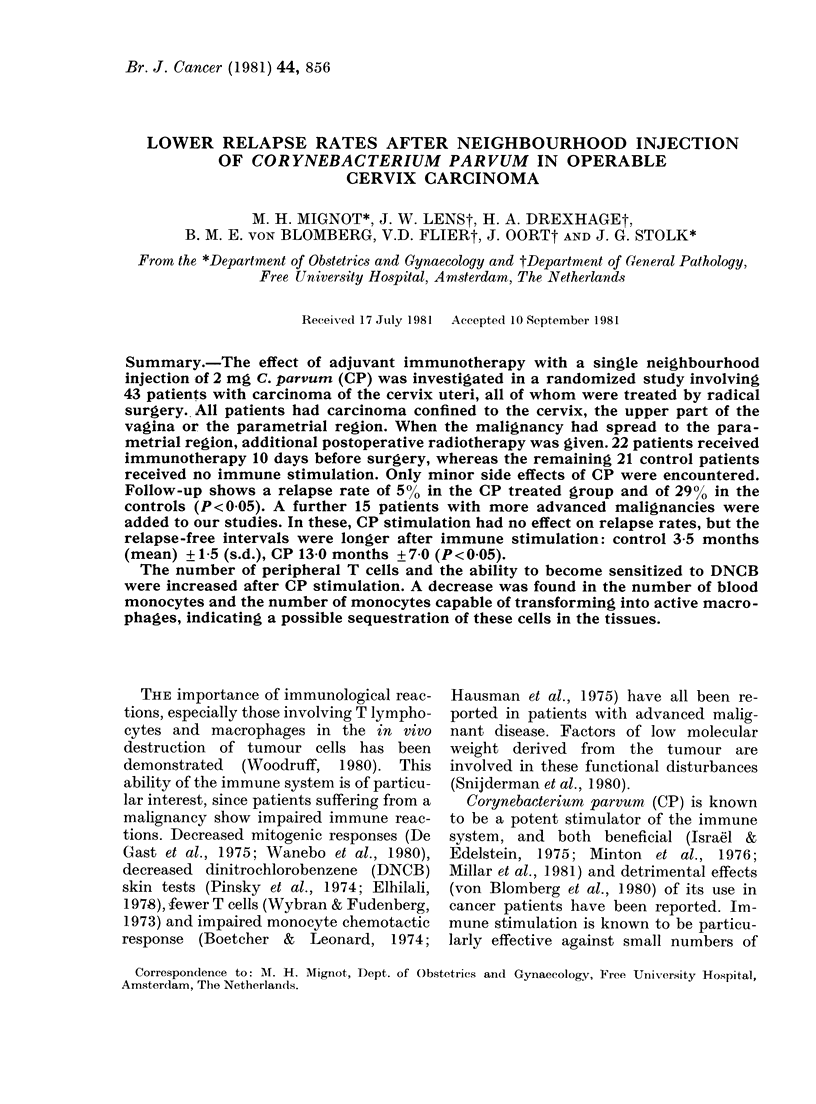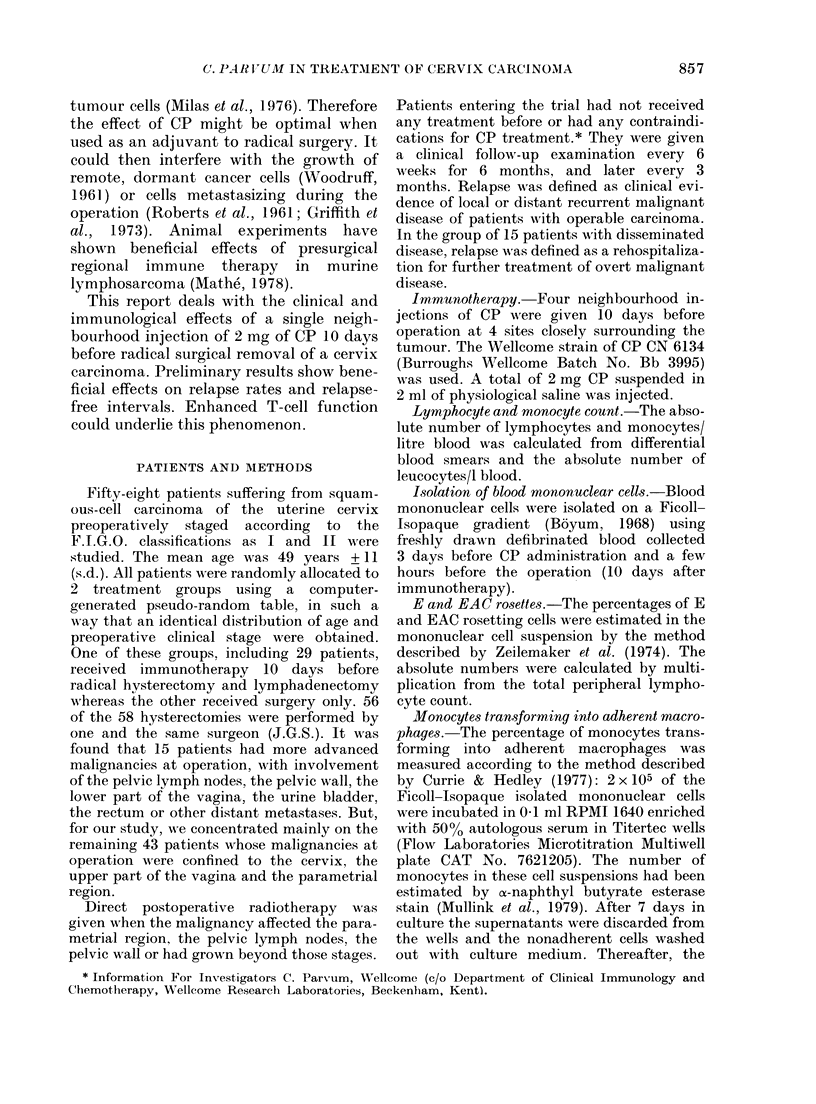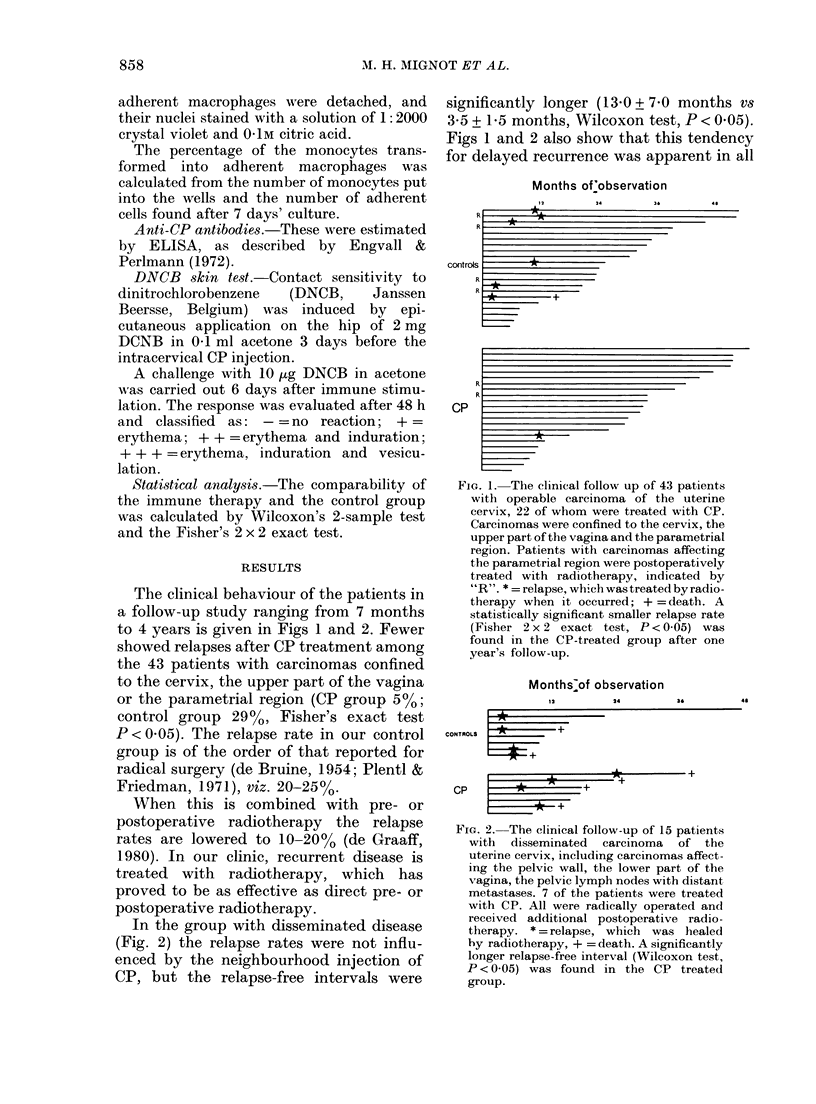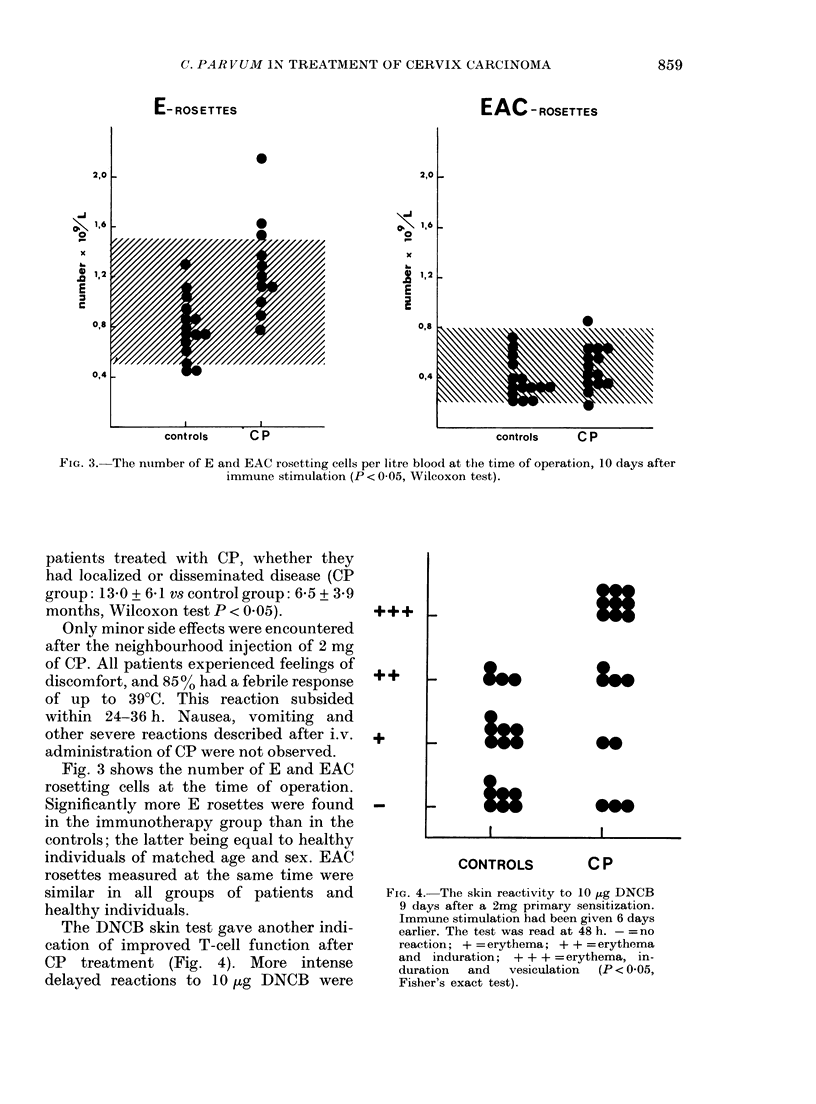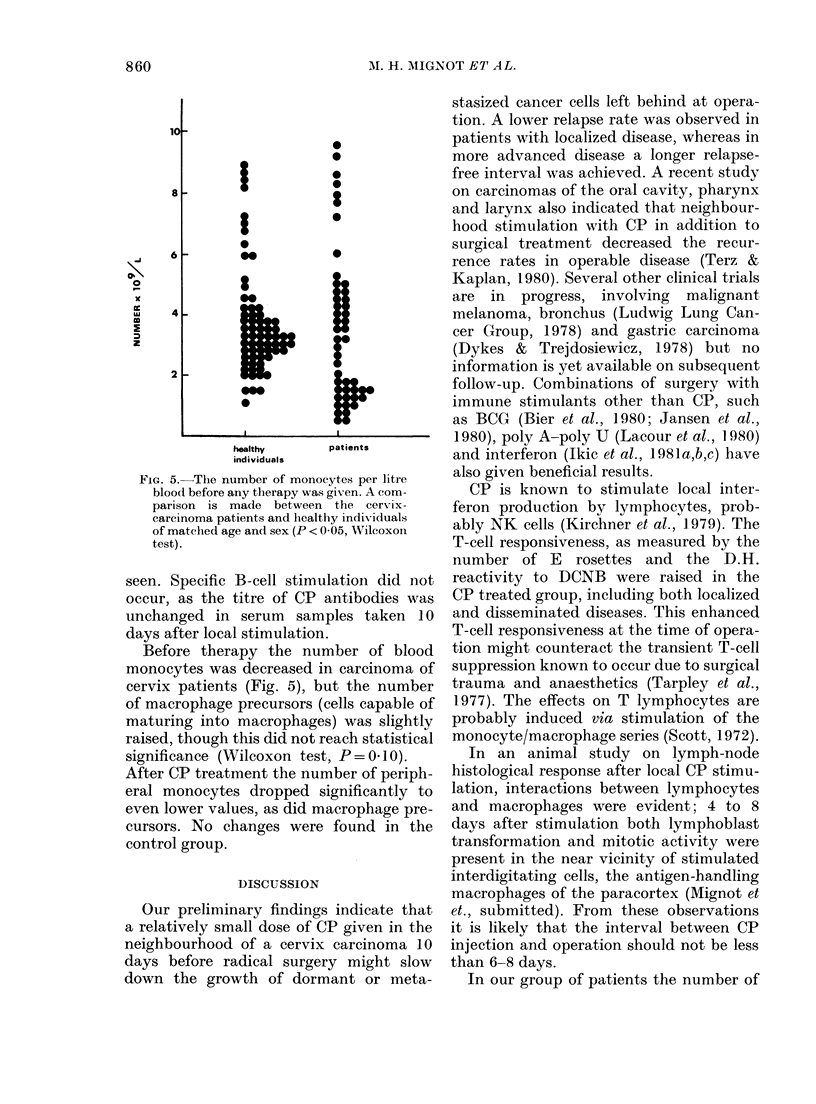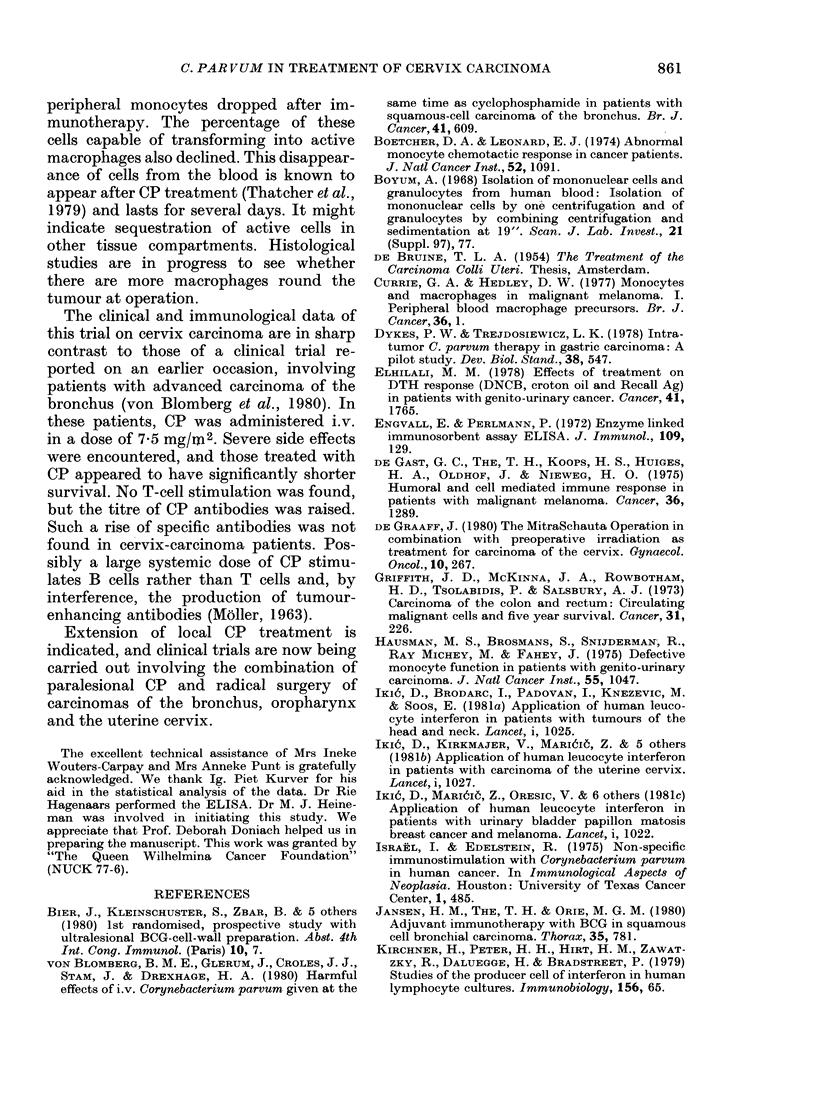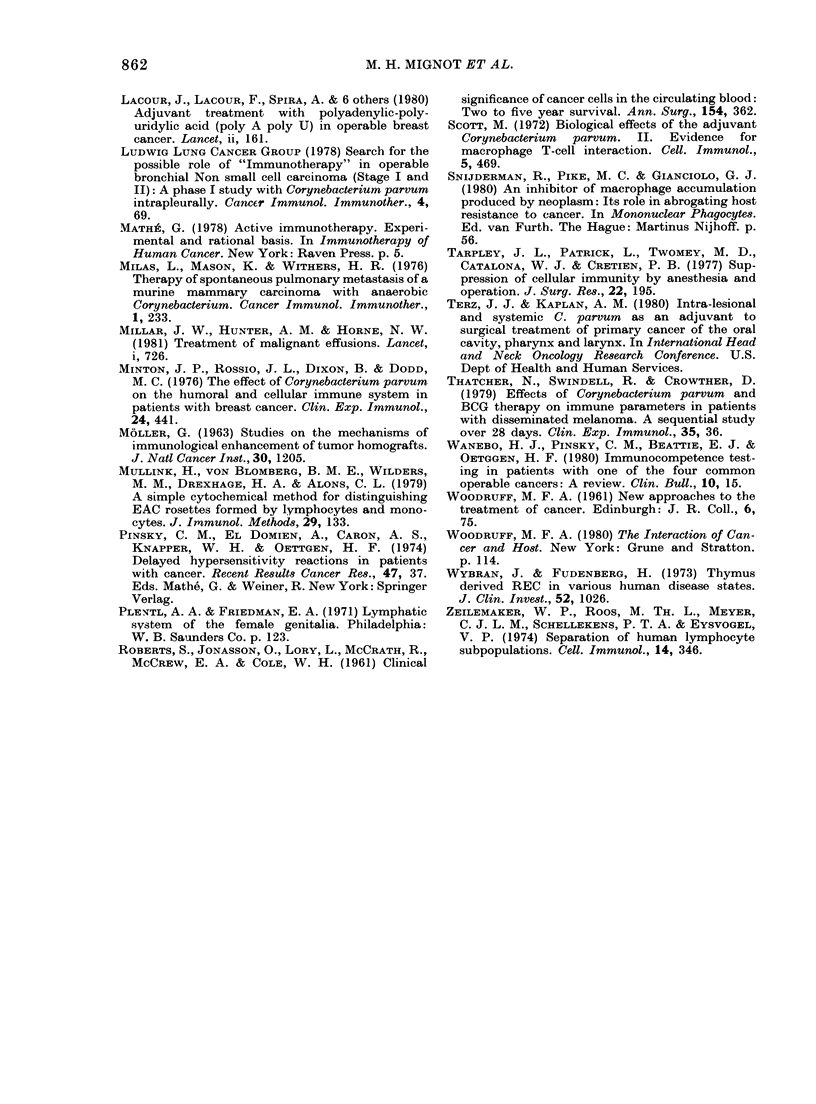# Lower relapse rates after neighbourhood injection of Corynebacterium parvum in operable cervix carcinoma.

**DOI:** 10.1038/bjc.1981.284

**Published:** 1981-12

**Authors:** M. H. Mignot, J. W. Lens, H. A. Drexhage, B. M. von Blomberg, V. D. Flier, J. Oort, J. G. Stolk

## Abstract

The effect of adjuvant immunotherapy with a single neighbourhood injection of 2 mg C. parvum (CP) was investigated in a randomized study involving 43 patients with carcinoma of the cervix uteri, all of whom were treated by radical surgery. All patients had carcinoma confined to the cervix, the upper part of the vagina or the parametrial region. When the malignancy had spread to the parametrial region, additional postoperative radiotherapy was given. 22 patients received immunotherapy 10 days before surgery, whereas the remaining 21 control patients received no immune stimulation. Only minor side effects of CP were encountered. Follow-up shows a relapse rate of 5% in the CP treated group and of 29% in the controls (P less than 0.05). A further 15 patients with more advanced malignancies were added to our studies. In these, CP stimulation had no effect on relapse rates, but the relapse-free intervals were longer after immune stimulation: control 3.5 months (mean) +/- 1.5 (s.d.), CP 13.0 months +/- 7.0 (P less than 0.05). The number of peripheral T cells and the ability to become sensitized to DNCB were increased after CP stimulation. A decrease was found in the number of blood monocytes and the number of monocytes capable of transforming into active macrophages, indicating a possible sequestration of these cells in the tissues.


*Br. J. Cancer* (1981) **44**, 856

### LOWER RELAPSE RATES AFTER NEIGHBOURHOOD INJECTION OF *CORYNEBACTERIUM PARVUM* IN OPERABLE CERVIX CARCINOMA

M. H. MIGNOT*, J. W. LENS†, H. A. DREXHAGE†,

B. M. E. VON BLOMBERG, V.D. FLIER†, J. OORT† AND J. G. STOLK*

*From the *Department of Obstetrics and Gynaecology and †Department of General Pathology, Free University Hospital, Amsterdam, The Netherlands*

Received 17 July 1981 Accepted 10 September 1981

**Summary.—The effect of adjuvant immunotherapy with a single neighbourhood injection of 2 mg *C. parvum* (CP) was investigated in a randomized study involving 43 patients with carcinoma of the cervix uteri, all of whom were treated by radical surgery. All patients had carcinoma confined to the cervix, the upper part of the vagina or the parametrial region. When the malignancy had spread to the parametrial region, additional postoperative radiotherapy was given. 22 patients received immunotherapy 10 days before surgery, whereas the remaining 21 control patients received no immune stimulation. Only minor side effects of CP were encountered. Follow-up shows a relapse rate of 5% in the CP treated group and of 29% in the controls ($P<0{\cdot}05$). A further 15 patients with more advanced malignancies were added to our studies. In these, CP stimulation had no effect on relapse rates, but the relapse-free intervals were longer after immune stimulation: control 3·5 months (mean) $\pm 1{\cdot}5$ (s.d.), CP 13·0 months $\pm 7{\cdot}0$ ($P<0{\cdot}05$).**

**The number of peripheral T cells and the ability to become sensitized to DNCB were increased after CP stimulation. A decrease was found in the number of blood monocytes and the number of monocytes capable of transforming into active macrophages, indicating a possible sequestration of these cells in the tissues.**

THE importance of immunological reactions, especially those involving T lymphocytes and macrophages in the *in vivo* destruction of tumour cells has been demonstrated (Woodruff, 1980). This ability of the immune system is of particular interest, since patients suffering from a malignancy show impaired immune reactions. Decreased mitogenic responses (De Gast *et al.*, 1975; Wanebo *et al.*, 1980), decreased dinitrochlorobenzene (DNCB) skin tests (Pinsky *et al.*, 1974; Elhilali, 1978), fewer T cells (Wybran & Fudenberg, 1973) and impaired monocyte chemotactic response (Boetcher & Leonard, 1974; Hausman *et al.*, 1975) have all been reported in patients with advanced malignant disease. Factors of low molecular weight derived from the tumour are involved in these functional disturbances (Snijderman *et al.*, 1980).

*Corynebacterium parvum* (CP) is known to be a potent stimulator of the immune system, and both beneficial (Israël & Edelstein, 1975; Minton *et al.*, 1976; Millar *et al.*, 1981) and detrimental effects (von Blomberg *et al.*, 1980) of its use in cancer patients have been reported. Immune stimulation is known to be particularly effective against small numbers of

Correspondence to: M. H. Mignot, Dept. of Obstetrics and Gynaecology, Free University Hospital, Amsterdam, The Netherlands.

tumour cells (Milas *et al.*, 1976). Therefore the effect of CP might be optimal when used as an adjuvant to radical surgery. It could then interfere with the growth of remote, dormant cancer cells (Woodruff, 1961) or cells metastasizing during the operation (Roberts *et al.*, 1961; Griffith *et al.*, 1973). Animal experiments have shown beneficial effects of presurgical regional immune therapy in murine lymphosarcoma (Mathé, 1978).

This report deals with the clinical and immunological effects of a single neighbourhood injection of 2 mg of CP 10 days before radical surgical removal of a cervix carcinoma. Preliminary results show beneficial effects on relapse rates and relapse-free intervals. Enhanced T-cell function could underlie this phenomenon.

### PATIENTS AND METHODS

Fifty-eight patients suffering from squamous-cell carcinoma of the uterine cervix preoperatively staged according to the F.I.G.O. classifications as I and II were studied. The mean age was 49 years $\pm 11$ (s.d.). All patients were randomly allocated to 2 treatment groups using a computer-generated pseudo-random table, in such a way that an identical distribution of age and preoperative clinical stage were obtained. One of these groups, including 29 patients, received immunotherapy 10 days before radical hysterectomy and lymphadenectomy whereas the other received surgery only. 56 of the 58 hysterectomies were performed by one and the same surgeon (J.G.S.). It was found that 15 patients had more advanced malignancies at operation, with involvement of the pelvic lymph nodes, the pelvic wall, the lower part of the vagina, the urine bladder, the rectum or other distant metastases. But, for our study, we concentrated mainly on the remaining 43 patients whose malignancies at operation were confined to the cervix, the upper part of the vagina and the parametrial region.

Direct postoperative radiotherapy was given when the malignancy affected the parametrial region, the pelvic lymph nodes, the pelvic wall or had grown beyond those stages. Patients entering the trial had not received any treatment before or had any contraindications for CP treatment.* They were given a clinical follow-up examination every 6 weeks for 6 months, and later every 3 months. Relapse was defined as clinical evidence of local or distant recurrent malignant disease of patients with operable carcinoma. In the group of 15 patients with disseminated disease, relapse was defined as a rehospitalization for further treatment of overt malignant disease.

*Immunotherapy.*—Four neighbourhood injections of CP were given 10 days before operation at 4 sites closely surrounding the tumour. The Wellcome strain of CP CN 6134 (Burroughs Wellcome Batch No. Bb 3995) was used. A total of 2 mg CP suspended in 2 ml of physiological saline was injected.

*Lymphocyte and monocyte count.*—The absolute number of lymphocytes and monocytes/litre blood was calculated from differential blood smears and the absolute number of leucocytes/l blood.

*Isolation of blood mononuclear cells.*—Blood mononuclear cells were isolated on a Ficoll–Isopaque gradient (Böyum, 1968) using freshly drawn defibrinated blood collected 3 days before CP administration and a few hours before the operation (10 days after immunotherapy).

*E and EAC rosettes.*—The percentages of E and EAC rosetting cells were estimated in the mononuclear cell suspension by the method described by Zeilemaker *et al.* (1974). The absolute numbers were calculated by multiplication from the total peripheral lymphocyte count.

*Monocytes transforming into adherent macrophages.*—The percentage of monocytes transforming into adherent macrophages was measured according to the method described by Currie & Hedley (1977): $2\times10^5$ of the Ficoll–Isopaque isolated mononuclear cells were incubated in 0·1 ml RPMI 1640 enriched with 50% autologous serum in Titertec wells (Flow Laboratories Microtitration Multiwell plate CAT No. 7621205). The number of monocytes in these cell suspensions had been estimated by $\alpha$-naphthyl butyrate esterase stain (Mullink *et al.*, 1979). After 7 days in culture the supernatants were discarded from the wells and the nonadherent cells washed out with culture medium. Thereafter, the

* Information For Investigators C. Parvum, Wellcome (c/o Department of Clinical Immunology and Chemotherapy, Wellcome Research Laboratories, Beckenham, Kent).

adherent macrophages were detached, and their nuclei stained with a solution of 1:2000 crystal violet and 0·1M citric acid.

The percentage of the monocytes transformed into adherent macrophages was calculated from the number of monocytes put into the wells and the number of adherent cells found after 7 days' culture.

*Anti-CP antibodies.*—These were estimated by ELISA, as described by Engvall & Perlmann (1972).

*DNCB skin test.*—Contact sensitivity to dinitrochlorobenzene (DNCB, Janssen Beersse, Belgium) was induced by epicutaneous application on the hip of 2 mg DCNB in 0·1 ml acetone 3 days before the intracervical CP injection.

A challenge with 10 μg DNCB in acetone was carried out 6 days after immune stimulation. The response was evaluated after 48 h and classified as: − = no reaction; + = erythema; + + = erythema and induration; + + + = erythema, induration and vesiculation.

*Statistical analysis.*—The comparability of the immune therapy and the control group was calculated by Wilcoxon's 2-sample test and the Fisher's 2 × 2 exact test.

### RESULTS

The clinical behaviour of the patients in a follow-up study ranging from 7 months to 4 years is given in Figs 1 and 2. Fewer showed relapses after CP treatment among the 43 patients with carcinomas confined to the cervix, the upper part of the vagina or the parametrial region (CP group 5%; control group 29%, Fisher's exact test $P < 0{\cdot}05$). The relapse rate in our control group is of the order of that reported for radical surgery (de Bruine, 1954; Plentl & Friedman, 1971), *viz.* 20–25%.

When this is combined with pre- or postoperative radiotherapy the relapse rates are lowered to 10–20% (de Graaff, 1980). In our clinic, recurrent disease is treated with radiotherapy, which has proved to be as effective as direct pre- or postoperative radiotherapy.

In the group with disseminated disease (Fig. 2) the relapse rates were not influenced by the neighbourhood injection of CP, but the relapse-free intervals were significantly longer ($13{\cdot}0 \pm 7{\cdot}0$ months *vs* $3{\cdot}5 \pm 1{\cdot}5$ months, Wilcoxon test, $P < 0{\cdot}05$). Figs 1 and 2 also show that this tendency for delayed recurrence was apparent in all

FIG. 1.—The clinical follow up of 43 patients with operable carcinoma of the uterine cervix, 22 of whom were treated with CP. Carcinomas were confined to the cervix, the upper part of the vagina and the parametrial region. Patients with carcinomas affecting the parametrial region were postoperatively treated with radiotherapy, indicated by "R". * = relapse, which was treated by radiotherapy when it occurred; + = death. A statistically significant smaller relapse rate (Fisher 2 × 2 exact test, $P < 0{\cdot}05$) was found in the CP-treated group after one year's follow-up.

FIG. 2.—The clinical follow-up of 15 patients with disseminated carcinoma of the uterine cervix, including carcinomas affecting the pelvic wall, the lower part of the vagina, the pelvic lymph nodes with distant metastases. 7 of the patients were treated with CP. All were radically operated and received additional postoperative radiotherapy. * = relapse, which was healed by radiotherapy, + = death. A significantly longer relapse-free interval (Wilcoxon test, $P < 0{\cdot}05$) was found in the CP treated group.

FIG. 3.—The number of E and EAC rosetting cells per litre blood at the time of operation, 10 days after immune stimulation ($P < 0·05$, Wilcoxon test).

patients treated with CP, whether they had localized or disseminated disease (CP group: $13·0 \pm 6·1$ *vs* control group: $6·5 \pm 3·9$ months, Wilcoxon test $P < 0·05$).

Only minor side effects were encountered after the neighbourhood injection of 2 mg of CP. All patients experienced feelings of discomfort, and 85% had a febrile response of up to 39°C. This reaction subsided within 24–36 h. Nausea, vomiting and other severe reactions described after i.v. administration of CP were not observed.

Fig. 3 shows the number of E and EAC rosetting cells at the time of operation. Significantly more E rosettes were found in the immunotherapy group than in the controls; the latter being equal to healthy individuals of matched age and sex. EAC rosettes measured at the same time were similar in all groups of patients and healthy individuals.

The DNCB skin test gave another indication of improved T-cell function after CP treatment (Fig. 4). More intense delayed reactions to 10 $\mu$g DNCB were

FIG. 4.—The skin reactivity to 10 $\mu$g DNCB 9 days after a 2mg primary sensitization. Immune stimulation had been given 6 days earlier. The test was read at 48 h. – = no reaction; + = erythema; + + = erythema and induration; + + + = erythema, induration and vesiculation ($P < 0·05$, Fisher's exact test).

FIG. 5.—The number of monocytes per litre blood before any therapy was given. A comparison is made between the cervix-carcinoma patients and healthy individuals of matched age and sex ($P < 0{\cdot}05$, Wilcoxon test).

seen. Specific B-cell stimulation did not occur, as the titre of CP antibodies was unchanged in serum samples taken 10 days after local stimulation.

Before therapy the number of blood monocytes was decreased in carcinoma of cervix patients (Fig. 5), but the number of macrophage precursors (cells capable of maturing into macrophages) was slightly raised, though this did not reach statistical significance (Wilcoxon test, $P = 0{\cdot}10$). After CP treatment the number of peripheral monocytes dropped significantly to even lower values, as did macrophage precursors. No changes were found in the control group.

### DISCUSSION

Our preliminary findings indicate that a relatively small dose of CP given in the neighbourhood of a cervix carcinoma 10 days before radical surgery might slow down the growth of dormant or metastasized cancer cells left behind at operation. A lower relapse rate was observed in patients with localized disease, whereas in more advanced disease a longer relapse-free interval was achieved. A recent study on carcinomas of the oral cavity, pharynx and larynx also indicated that neighbourhood stimulation with CP in addition to surgical treatment decreased the recurrence rates in operable disease (Terz & Kaplan, 1980). Several other clinical trials are in progress, involving malignant melanoma, bronchus (Ludwig Lung Cancer Group, 1978) and gastric carcinoma (Dykes & Trejdosiewicz, 1978) but no information is yet available on subsequent follow-up. Combinations of surgery with immune stimulants other than CP, such as BCG (Bier *et al.*, 1980; Jansen *et al.*, 1980), poly A–poly U (Lacour *et al.*, 1980) and interferon (Ikic *et al.*, 1981*a,b,c*) have also given beneficial results.

CP is known to stimulate local interferon production by lymphocytes, probably NK cells (Kirchner *et al.*, 1979). The T-cell responsiveness, as measured by the number of E rosettes and the D.H. reactivity to DCNB were raised in the CP treated group, including both localized and disseminated diseases. This enhanced T-cell responsiveness at the time of operation might counteract the transient T-cell suppression known to occur due to surgical trauma and anaesthetics (Tarpley *et al.*, 1977). The effects on T lymphocytes are probably induced *via* stimulation of the monocyte/macrophage series (Scott, 1972).

In an animal study on lymph-node histological response after local CP stimulation, interactions between lymphocytes and macrophages were evident; 4 to 8 days after stimulation both lymphoblast transformation and mitotic activity were present in the near vicinity of stimulated interdigitating cells, the antigen-handling macrophages of the paracortex (Mignot *et et.*, submitted). From these observations it is likely that the interval between CP injection and operation should not be less than 6–8 days.

In our group of patients the number of peripheral monocytes dropped after immunotherapy. The percentage of these cells capable of transforming into active macrophages also declined. This disappearance of cells from the blood is known to appear after CP treatment (Thatcher *et al.*, 1979) and lasts for several days. It might indicate sequestration of active cells in other tissue compartments. Histological studies are in progress to see whether there are more macrophages round the tumour at operation.

The clinical and immunological data of this trial on cervix carcinoma are in sharp contrast to those of a clinical trial reported on an earlier occasion, involving patients with advanced carcinoma of the bronchus (von Blomberg *et al.*, 1980). In these patients, CP was administered i.v. in a dose of 7·5 mg/m$^2$. Severe side effects were encountered, and those treated with CP appeared to have significantly shorter survival. No T-cell stimulation was found, but the titre of CP antibodies was raised. Such a rise of specific antibodies was not found in cervix-carcinoma patients. Possibly a large systemic dose of CP stimulates B cells rather than T cells and, by interference, the production of tumour-enhancing antibodies (Möller, 1963).

Extension of local CP treatment is indicated, and clinical trials are now being carried out involving the combination of paralesional CP and radical surgery of carcinomas of the bronchus, oropharynx and the uterine cervix.

The excellent technical assistance of Mrs Ineke Wouters-Carpay and Mrs Anneke Punt is gratefully acknowledged. We thank Ig. Piet Kurver for his aid in the statistical analysis of the data. Dr Rie Hagenaars performed the ELISA. Dr M. J. Heineman was involved in initiating this study. We appreciate that Prof. Deborah Doniach helped us in preparing the manuscript. This work was granted by "The Queen Wilhelmina Cancer Foundation" (NUCK 77-6).

### REFERENCES

BIER, J., KLEINSCHUSTER, S., ZBAR, B. & 5 others (1980) 1st randomised, prospective study with ultralesional BCG-cell-wall preparation. *Abst. 4th Int. Cong. Immunol.* (Paris) **10**, 7.

VON BLOMBERG, B. M. E., GLERUM, J., CROLES, J. J., STAM, J. & DREXHAGE, H. A. (1980) Harmful effects of i.v. *Corynebacterium parvum* given at the same time as cyclophosphamide in patients with squamous-cell carcinoma of the bronchus. *Br. J. Cancer*, **41**, 609.

BOETCHER, D. A. & LEONARD, E. J. (1974) Abnormal monocyte chemotactic response in cancer patients. *J. Natl Cancer Inst.*, **52**, 1091.

BOYUM, A. (1968) Isolation of mononuclear cells and granulocytes from human blood: Isolation of mononuclear cells by one centrifugation and of granulocytes by combining centrifugation and sedimentation at 19''. *Scan. J. Lab. Invest.*, **21** (Suppl. 97), 77.

DE BRUINE, T. L. A. (1954) *The Treatment of the Carcinoma Colli Uteri.* Thesis, Amsterdam.

CURRIE, G. A. & HEDLEY, D. W. (1977) Monocytes and macrophages in malignant melanoma. I. Peripheral blood macrophage precursors. *Br. J. Cancer*, **36**, 1.

DYKES, P. W. & TREJDOSIEWICZ, L. K. (1978) Intratumor *C. parvum* therapy in gastric carcinoma: A pilot study. *Dev. Biol. Stand.*, **38**, 547.

ELHILALI, M. M. (1978) Effects of treatment on DTH response (DNCB, croton oil and Recall Ag) in patients with genito-urinary cancer. *Cancer*, **41**, 1765.

ENGVALL, E. & PERLMANN, P. (1972) Enzyme linked immunosorbent assay ELISA. *J. Immunol.*, **109**, 129.

DE GAST, G. C., THE, T. H., KOOPS, H. S., HUIGES, H. A., OLDHOF, J. & NIEWEG, H. O. (1975) Humoral and cell mediated immune response in patients with malignant melanoma. *Cancer*, **36**, 1289.

DE GRAAFF, J. (1980) The MitraSchauta Operation in combination with preoperative irradiation as treatment for carcinoma of the cervix. *Gynaecol. Oncol.*, **10**, 267.

GRIFFITH, J. D., MCKINNA, J. A., ROWBOTHAM, H. D., TSOLABIDIS, P. & SALSBURY, A. J. (1973) Carcinoma of the colon and rectum: Circulating malignant cells and five year survival. *Cancer*, **31**, 226.

HAUSMAN, M. S., BROSMANS, S., SNIJDERMAN, R., RAY MICHEY, M. & FAHEY, J. (1975) Defective monocyte function in patients with genito-urinary carcinoma. *J. Natl Cancer Inst.*, **55**, 1047.

IKIĆ, D., BRODARC, I., PADOVAN, I., KNEZEVIC, M. & SOOS, E. (1981*a*) Application of human leucocyte interferon in patients with tumours of the head and neck. *Lancet*, i, 1025.

IKIĆ, D., KIRKMAJER, V., MARIĆIČ, Z. & 5 others (1981*b*) Application of human leucocyte interferon in patients with carcinoma of the uterine cervix. *Lancet*, i, 1027.

IKIĆ, D., MARIĆIČ, Z., ORESIC, V. & 6 others (1981c) Application of human leucocyte interferon in patients with urinary bladder papillon matosis breast cancer and melanoma. *Lancet*, i, 1022.

ISRAËL, I. & EDELSTEIN, R. (1975) Non-specific immunostimulation with *Corynebacterium parvum* in human cancer. In *Immunological Aspects of Neoplasia.* Houston: University of Texas Cancer Center, **1**, 485.

JANSEN, H. M., THE, T. H. & ORIE, M. G. M. (1980) Adjuvant immunotherapy with BCG in squamous cell bronchial carcinoma. *Thorax*, **35**, 781.

KIRCHNER, H., PETER, H. H., HIRT, H. M., ZAWATZKY, R., DALUEGGE, H. & BRADSTREET, P. (1979) Studies of the producer cell of interferon in human lymphocyte cultures. *Immunobiology*, **156**, 65.

LACOUR, J., LACOUR, F., SPIRA, A. & 6 others (1980) Adjuvant treatment with polyadenylic-polyuridylic acid (poly A poly U) in operable breast cancer. *Lancet*, ii, 161.

LUDWIG LUNG CANCER GROUP (1978) Search for the possible role of "Immunotherapy" in operable bronchial Non small cell carcinoma (Stage I and II): A phase I study with *Corynebacterium parvum* intrapleurally. *Cancer Immunol. Immunother.*, **4**, 69.

MATHÉ, G. (1978) Active immunotherapy. Experimental and rational basis. In *Immunotherapy of Human Cancer*. New York: Raven Press. p. 5.

MILAS, L., MASON, K. & WITHERS, H. R. (1976) Therapy of spontaneous pulmonary metastasis of a murine mammary carcinoma with anaerobic *Corynebacterium. Cancer Immunol. Immunother.*, **1**, 233.

MILLAR, J. W., HUNTER, A. M. & HORNE, N. W. (1981) Treatment of malignant effusions. *Lancet*, i, 726.

MINTON, J. P., ROSSIO, J. L., DIXON, B. & DODD, M. C. (1976) The effect of *Corynebacterium parvum* on the humoral and cellular immune system in patients with breast cancer. *Clin. Exp. Immunol.*, **24**, 441.

MÖLLER, G. (1963) Studies on the mechanisms of immunological enhancement of tumor homografts. *J. Natl Cancer Inst.*, **30**, 1205.

MULLINK, H., VON BLOMBERG, B. M. E., WILDERS, M. M., DREXHAGE, H. A. & ALONS, C. L. (1979) A simple cytochemical method for distinguishing EAC rosettes formed by lymphocytes and monocytes. *J. Immunol. Methods*, **29**, 133.

PINSKY, C. M., EL DOMIEN, A., CARON, A. S., KNAPPER, W. H. & OETTGEN, H. F. (1974) Delayed hypersensitivity reactions in patients with cancer. *Recent Results Cancer Res.*, **47**, 37. Eds. Mathé, G. & Weiner, R. New York: Springer Verlag.

PLENTL, A. A. & FRIEDMAN, E. A. (1971) Lymphatic system of the female genitalia. Philadelphia: W. B. Saunders Co. p. 123.

ROBERTS, S., JONASSON, O., LORY, L., MCCRATH, R., MCCREW, E. A. & COLE, W. H. (1961) Clinical significance of cancer cells in the circulating blood: Two to five year survival. *Ann. Surg.*, **154**, 362.

SCOTT, M. (1972) Biological effects of the adjuvant *Corynebacterium parvum*. II. Evidence for macrophage T-cell interaction. *Cell. Immunol.*, **5**, 469.

SNIJDERMAN, R., PIKE, M. C. & GIANCIOLO, G. J. (1980) An inhibitor of macrophage accumulation produced by neoplasm: Its role in abrogating host resistance to cancer. In *Mononuclear Phagocytes*. Ed. van Furth. The Hague: Martinus Nijhoff. p. 56.

TARPLEY, J. L., PATRICK, L., TWOMEY, M. D., CATALONA, W. J. & CRETIEN, P. B. (1977) Suppression of cellular immunity by anesthesia and operation. *J. Surg. Res.*, **22**, 195.

TERZ, J. J. & KAPLAN, A. M. (1980) Intra-lesional and systemic *C. parvum* as an adjuvant to surgical treatment of primary cancer of the oral cavity, pharynx and larynx. In *International Head and Neck Oncology Research Conference*. U.S. Dept of Health and Human Services.

THATCHER, N., SWINDELL, R. & CROWTHER, D. (1979) Effects of *Corynebacterium parvum* and BCG therapy on immune parameters in patients with disseminated melanoma. A sequential study over 28 days. *Clin. Exp. Immunol.*, **35**, 36.

WANEBO, H. J., PINSKY, C. M., BEATTIE, E. J. & OETGGEN, H. F. (1980) Immunocompetence testing in patients with one of the four common operable cancers: A review. *Clin. Bull.*, **10**, 15.

WOODRUFF, M. F. A. (1961) New approaches to the treatment of cancer. Edinburgh: J. R. Coll., **6**, 75.

WOODRUFF, M. F. A. (1980) *The Interaction of Cancer and Host*. New York: Grune and Stratton. p. 114.

WYBRAN, J. & FUDENBERG, H. (1973) Thymus derived REC in various human disease states. *J. Clin. Invest.*, **52**, 1026.

ZEILEMAKER, W. P., ROOS, M. TH. L., MEYER, C. J. L. M., SCHELLEKENS, P. T. A. & EYSVOGEL, V. P. (1974) Separation of human lymphocyte subpopulations. *Cell. Immunol.*, **14**, 346.